# „Brain fog“ in den Wechseljahren

**DOI:** 10.1007/s10304-022-00488-w

**Published:** 2023-01-02

**Authors:** Petra Stute

**Affiliations:** Gynäkologische Endokrinologie und Reproduktionsmedizin, Inselspital Bern, Friedbühlstr. 19, 3010 Bern, Schweiz

## Originalpublikation

Maki PM, Jaff NG (2022) Brain fog in menopause: a health-care professional’s guide for decision-making and counseling on cognition. Climacteric 30:1–9. 10.1080/13697137.2022.2122792

## Hintergrund.

Kognitive Beschwerden sind in den Wechseljahren häufig und mit einer reduzierten Lebensqualität verbunden [1]. Spätestens seit der Pandemie aufgrund von SARS-CoV-2(„severe acute respiratory syndrome coronavirus 2“)-Infektionen ist den meisten der Begriff „brain fog“ geläufig, der nun auch zunehmend im Kontext der Wechseljahre gebraucht wird. Wie sollten Frauen mit „brain fog“ in den Wechseljahren beraten werden?

## Zusammenfassung.

Unter „brain fog“ in den Wechseljahren versteht man verschiedene kognitive Symptome, die sich häufig als Schwierigkeiten im Gedächtnis und in der Aufmerksamkeit manifestieren. Diese kognitiven Veränderungen in den Wechseljahren sollten nicht mit einer Demenz verwechselt werden. Eine Demenz in einem Alter unter 64 Jahren ist selten. Zu den häufigsten kognitiven Beschwerden zählen Schwierigkeiten beim Lernen und im verbalen Gedächtnis. Die Symptome beginnen meist während der menopausalen Transition. Die Beschwerden können störend und subjektiv besorgniserregend sein, der normale kognitive Funktionsumfang wird jedoch in der Regel beibehalten; nur etwa 11–13 % der Frauen weisen eine klinisch signifikante Beeinträchtigung auf. Die kognitiven Symptome sind mit Veränderungen der Östrogenserumkonzentration, vasomotorischen Beschwerden, Schlaf und Stimmung assoziiert. Die Behandlung dieser Symptome kann sich positiv auf die Kognition auswirken. Es stellt sich die grundsätzliche Frage nach der Rolle der Hormonersatztherapie („hormone replacement therapy“, HRT) im Hinblick auf Kognition und Demenz. Eine HRT wird derzeit international weder zur Behandlung kognitiver Beschwerden in den Wechseljahren noch zur Prävention eines kognitiven Abbaus bzw. einer Demenzentwicklung empfohlen. Fragen zum Einfluss einer HRT auf die kognitiven Fähigkeiten bei Frauen mit störenden Hitzewallungen oder bei Frauen in der Perimenopause können mangels entsprechender Studien noch nicht beantwortet werden. Bei Frauen mit früher Menopause (< 45 Jahre) unterstützen Östrogene den Erhalt der kognitiven Funktion und reduzieren das Demenzrisiko. Wenn eine HRT in der frühen Postmenopause begonnen wird, ist kein negativer Einfluss auf die Kognition zu erwarten. Gleiches gilt für den Einsatz von reinen Östrogenen in der späten Postmenopause. Bisher konnte nur für die kombinierte HRT mit konjugierten equinen Östrogenen (CEE) und Medroxyprogesteronacetat (MPA) ein negativer Einfluss auf die kognitive Funktion beobachtet werden, wenn diese mit 65 Jahren gestartet (!) wird. Die Kombination von oralem Estradiol und vaginalem Progesteron scheint dagegen auch bei Start in der späten Postmenopause sicher zu sein. Verschiedene modifizierbare Risikofaktoren sind mit der kognitiven Gesundheit assoziiert. Hierzu zählen:

- Adipositas,
- Bluthochdruck,
- Diabetes,
- mangelnde körperliche Aktivität,
- Rauchen,
- mangelnde kognitive Aktivität,
- wenig soziale Interaktion,
- Schwerhörigkeit,
- Depression.

Diese Risikofaktoren sollten möglichst gut gemanagt/reduziert werden. Es liegen bisher keine ausreichenden Daten vor, um zwischen kognitiven Beschwerden aufgrund der Wechseljahre und aufgrund von Long-COVID („coronavirus disease“) unterscheiden zu können. Allerdings scheinen exekutive Funktionseinbußen, welche in der Regel in den Wechseljahren nicht beobachtet werden, bei SARS-CoV‑2 ein herausragendes Merkmal zu sein.

## Kommentar

Der Review bietet praktische Empfehlungen zum Management von Frauen mit kognitiven Beschwerden in den Wechseljahren. Am wichtigsten ist, dass diese kognitiven Beschwerden nicht, wie häufig befürchtet, einen Vorboten der Demenz darstellen. Auch das Demenzrisiko unter HRT wird aufgegriffen, und es wird verdeutlicht, dass ein erhöhtes Risiko bisher nur für asymptomatische (!) mindestens 65-jährige (!) Frauen beobachtet wurde, die erst zu diesem Zeitpunkt (!) mit einer oralen kombinierten HRT, bestehend aus CEE und MPA (!), beginnen [2].
